# Integrating single-cell analysis and machine learning algorithms to explore lactylation-related molecular mechanisms and therapeutic responses in clear cell renal cell carcinoma and identifying CDT1 as a potential biomarker

**DOI:** 10.3389/fendo.2026.1883553

**Published:** 2026-08-06

**Authors:** Yue Zhang, Hang Zhou, Bihui Zhang, Xianchao Sun, Wangli Mei, Lin Zhou

**Affiliations:** 1 Department of Laboratory Medicine, Shanghai Changzheng Hospital, Naval Medical University, Shanghai, China; 2 Department of Urology, Shanghai East Hospital, School of Medicine, Tongji University, Shanghai, China; 3 Urologic Cancer Institute, School of Medicine, Tongji University, Shanghai, China; 4 Department of Urology, Shanghai Fourth People’s Hospital, School of Medicine, Tongji University, Shanghai, China; 5 Department of Urology, The Second Affiliated Hospital of Anhui Medical University, Hefei, China

**Keywords:** biomarker, CDT1, lactylation, machine learning algorithms, renal cell carcinoma, single-cell analysis

## Abstract

**Background and aim:**

Lactylation is a novel histone modification driven by lactate accumulation, which has been implicated in clear cell renal cell carcinoma (ccRCC) progression. However, its comprehensive molecular mechanism and clinical relevance remain poorly understood. This study aimed to investigate lactylation-related molecular mechanisms and therapeutic responses in ccRCC using integrated single-cell analysis and machine learning algorithms.

**Methods:**

We integrated single-cell RNA sequencing and bulk transcriptomic data from patients with ccRCC. Transcriptional signatures of lactylation-related genes were evaluated using four gene set scoring algorithms. Key lactylation-related genes were identified through weighted gene co-expression network analysis and differential expression analysis. A prognostic model was constructed using 10 machine learning algorithms and subsequently validated in independent cohorts. The functional role of the core gene, *CDT1*, was validated through *in vitro* and *in vivo* experiments.

**Results:**

We established a prognostic model comprising 13 lactylation-related genes. The model robustly stratified patients into high- and low-risk groups with distinct survival outcomes, immune microenvironment features, and immunotherapy responses. Functional assays revealed that *CDT1*, the core gene of the signature, promoted ccRCC cell proliferation, migration, and invasion. Moreover, modulation of CDT1 altered intracellular L-lactate levels. However, whether this effect reflects a direct metabolic–epigenetic regulatory mechanism or is secondary to proliferative changes remains to be elucidated.

**Conclusions:**

This study delineates the molecular heterogeneity associated with lactylation-related gene expression in ccRCC and presents a validated prognostic model. It identifies *CDT1* as a novel oncogene and potential biomarker, providing insights into metabolic–epigenetic crosstalk and a foundation for future therapeutic development.

## Introduction

Renal cell carcinoma (RCC) is one of the most prevalent malignancies of the urinary system, with an estimated 431,288 new cases diagnosed globally in 2020 alone (1). Clear cell renal cell carcinoma (ccRCC), the predominant histological subtype, accounts for approximately 70% of all renal cancers (2). Although surgical resection offers a potentially curative treatment for localized tumors, the marked metabolic reprogramming and potent immune-evasive phenotype of ccRCC contribute to heterogeneous clinical presentation and suboptimal therapeutic responses (3). Somatic mutations and epigenetic silencing of the von Hippel–Lindau (VHL) tumor suppressor gene are among the most well-characterized and common genetic alterations in ccRCC (4, 5). However, accumulating evidence indicates that VHL inactivation is dispensable in a subset of patients, suggesting the existence of alternative oncogenic drivers (6). Consequently, the identification of novel biomarkers for ccRCC is essential for understanding the molecular mechanisms underlying tumor progression. Recent studies on epigenetics have underscored the pivotal role of posttranslational modifications (PTMs) in orchestrating tumor evolution and microenvironment remodeling (7). Among these, lactylation—a newly identified histone modification driven by lactate accumulation—has emerged as a key nexus linking metabolic dysregulation to transcriptional reprogramming in cancer cells (8, 9). Given the established Warburg effect in ccRCC, which induces excessive lactate production (5), investigating lactylation-mediated epigenetic alterations may yield transformative insights into ccRCC pathogenesis and refine patient stratification strategies.

Histone lysine lactylation (Kla), which was first identified in 2019, is a recently described histone modification in which a lactate-derived moiety is covalently conjugated to the ϵ-amino group of a lysine residue (10). Beyond canonical histones, Kla has been detected on multiple non-histone proteins, underscoring the breadth of these PTMs across the cellular proteome. The chemical basis of lactylation entails the covalent attachment of a lactyl group to target proteins, a process that proceeds through either enzymatic lactyl-CoA-dependent pathways or nonenzymatic mechanisms (11, 12). In ccRCC, glycolytic flux is markedly elevated compared with oxidative phosphorylation, which is a metabolic shift that drives substantial lactate accumulation within the tumor microenvironment. This accumulated lactate not only modulates diverse cellular physiological processes but also stabilizes HIF-1α to induce a myeloid-derived suppressor cell–like phenotype, thereby contributing to RCC prognosis (9). Lysine lactylation functions as a key nexus between cellular metabolism and transcriptional output, actively participating in the regulation of multiple oncogenic signaling cascades. Yang et al. (13) demonstrated that VHL inactivation triggered histone lactylation, which, in turn, activated the transcription of platelet-derived growth factor receptor β (PDGFRβ) and accelerated ccRCC progression. PDGFRβ signaling further reinforced histone lactylation, establishing a pro-tumorigenic positive feedback loop. Thus, a systematic investigation into the functional roles of lactylation-related genes in ccRCC may yield transformative insights into the metabolic regulation of tumor biology.

Advances in high-throughput sequencing have catalyzed a paradigm shift in transcriptomic analysis (14). In particular, single-cell transcriptomics now enables the deconvolution of tumor heterogeneity with unprecedented resolution, overcoming the limitations of bulk transcriptomic approaches and highlighting the cellular determinants of progression and therapeutic resistance in ccRCC. In this study, we first delineated the functional roles of lactylation-related genes in ccRCC through integrative single-cell RNA sequencing (scRNA-seq) and bulk transcriptomic analyses, followed by mechanistic validation using *in vitro a*nd *in vivo* models. We then constructed a lactylation-based prognostic model by systematically benchmarking 10 machine learning algorithms across 101 distinct algorithmic combinations and validated its predictive performance in an independent external validation cohort. Furthermore, we stratified patients based on their model-derived risk scores to examine immune microenvironmental differences across risk subgroups and forecast differential responses to immunotherapy. Collectively, this multi-omic, integrative framework aimed to improve our understanding of metabolic–epigenetic crosstalk in ccRCC and establish a robust molecular foundation for prognostic assessment and therapeutic decision-making.

## Methods

### Data collection and processing

scRNA-seq data were obtained from the GSE156632 dataset (https://www.ncbi.nlm.nih.gov/geo/, accessed on 2025-09-26), comprising samples from seven patients with ccRCC. Data preprocessing was performed using the Seurat R package (v5.2.1), including quality control and selection of the top 2000 highly variable genes. Batch effects were corrected using the Harmony R package (v 1.2.3). Dimensionality reduction was conducted using t-distributed stochastic neighbor embedding (t-SNE). A resolution of 0.3 was selected for subsequent clustering analysis.

Bulk RNA-seq data and corresponding clinical information for ccRCC were retrieved from The Cancer Genome Atlas (TCGA) database (https://portal.gdc.cancer.gov/, accessed on 2025-09-30). After excluding samples with incomplete clinical or prognostic records, 529 patients with transcripts per million–normalized RNA-seq expression data were included in the study. Single-nucleotide variant data were also obtained to assess tumor mutational burden. For external validation, RNA-seq and clinical data were downloaded from the E-MTAB-1980 (https://www.ebi.ac.uk/biostudies/arrayexpress/studies/E-MTAB-1980, accessed on 2025-09-30) and GSE29609 (https://www.ncbi.nlm.nih.gov/geo/, accessed on 2025-09-30) datasets. In addition, data from the IMvigor210 cohort were included to evaluate the predictive performance of the model for immunotherapy response.

### Single-cell data scoring using four algorithms

We compiled a set of 364 genes from previously published studies to identify lactylation-related genes (**Supplementary Table 1**). We systematically applied four gene set scoring algorithms to evaluate the expression patterns and functional relevance of lactylation-related gene sets in ccRCC at single-cell resolution: AUCell, UCell, AddModuleScore, and single-sample gene set enrichment analysis (ssGSEA) (15, 16). Each algorithm offers distinct advantages for single-cell analysis: AUCell detects subtle enrichment of gene sets at the expression level; UCell performs cell-level scoring independently of cell count and random seeds; AddModuleScore computes module scores by subtracting the average expression of background gene sets; and ssGSEA incorporates gene–gene interactions and functional associations to more accurately reflect the activation state of biological processes and pathways. Scores from these complementary approaches were integrated to cross-validate lactylation-associated transcriptional activity and mitigate bias inherent to any individual algorithm. A composite score for lactylation-related gene sets was calculated as the mean of all algorithm scores. The cells were then stratified into high- and low-score groups based on the median composite score across all cells. Marker genes in the high-score group were identified using the FindMarkers algorithm.

### Weighted gene co-expression network analysis

Weighted gene co-expression network analysis (WGCNA) was performed to identify gene modules associated with lactylation-related transcriptional scores. After sample clustering and outlier removal, a scale-free co-expression network was constructed based on gene expression variability. The adjacency matrix was converted into a topological overlap matrix to assess gene–gene network connectivity, and highly correlated modules were identified for further investigation. Lactylation-related genes identified from single-cell analysis were then integrated into the WGCNA framework to validate the functional relevance of these networks at the bulk transcriptomic level, enabling systematic screening and prioritization of key biological networks. Using TCGA patient data, this analysis encompassed data preprocessing, module detection, and functional annotation to comprehensively evaluate co-expression patterns and the potential biological significance of lactylation-related genes in the ccRCC transcriptome.

### Enrichment analysis

Differential expression analysis between tumor and normal samples in the TCGA-KIRC cohort was performed using the limma R package (v 3.58.1), with criteria of |logFC| >1 and *P* < 0.05. Lactylation-associated gene modules identified by WGCNA were then integrated with bulk-level differentially expressed genes (DEGs) to select genes consistently linked to lactylation across both bulk and single-cell transcriptomic contexts. These genes were defined as lactylation-related genes. Subsequently, intersecting these lactylation-related genes with DEGs yielded 15 hub genes. Kyoto Encyclopedia of Genes and Genomes (KEGG) and Gene Ontology (GO) enrichment analyses were performed using the clusterProfiler R package (v 4.10.1) to investigate the biological functions of these core genes.

We also performed gene set enrichment analysis (GSEA). Enrichment scores (ES) were calculated, and enrichment curves were generated to visualize the distribution of predefined gene sets across sample groups. ssGSEA was used to quantify pathway activity in individual samples. Furthermore, the GSVA R package (v 1.50.5) was used to compute lactylation scores for each ccRCC sample. GSVA ES for 50 feature pathways were analyzed using the limma package, and enrichment differences across these pathways were compared between risk groups.

### Development of the lactylation-related prognostic model

First, all model training and validation were performed using bulk transcriptome data rather than single-cell RNA sequencing data, which represented whole-tissue expression profiles of clinical tumor samples. Univariate Cox regression analysis was first performed in the TCGA-KIRC dataset to identify lactylation-related genes with potential prognostic value. The TCGA-KIRC cohort served as the training set, whereas the GSE29609 and E-MTAB-1980 datasets were used as independent external validation cohorts. To optimize prognostic modeling, 101 prognostic signatures were constructed using 10 machine learning algorithms: least absolute shrinkage and selection operator (LASSO), random survival forest (RSF), stepwise Cox regression, ridge regression, CoxBoost, elastic net (Enet), generalized boosted regression modeling, plsRcox, survival support vector machine, and supervised principal component analysis.

The predictive performance of each model was evaluated using the concordance index (C-index) in both the training and external validation sets, and all models were ranked according to their average C-index. Finally, we selected the algorithm combination demonstrating robust predictive stability and clinical applicability. We comprehensively evaluated these 10 algorithms and their 101 combinations, including regression-based methods (e.g., logistic regression, LASSO, and ridge regression) and ensemble models, to identify the most robust prognostic model. Given the relatively small feature set (15 genes), penalized algorithms (LASSO and ridge regression) were applied to reduce model redundancy and prevent overfitting, although the number of features removed was modest. Finally, by integrating RSF with Enet regression, we developed a lactylation-related prognostic signature comprising 13 key genes: *NR3C2*, *E2F1*, *ASF1B*, *CDT1*, *TPX2*, *CCNA2*, *TOP2A*, *PTTG1*, *LMNB1*, *MKI67*, *BIRC5*, *MYBL2*, and *PLAUR*.

The final prognostic model was developed using a two-step approach combining RSF and Enet (*α* = 0.1). First, RSF was applied to the TCGA training cohort to rank all candidate genes by variable importance. Genes with positive variable importance values were retained as the initial feature set. Second, Enet regression (*α* = 0.1) was performed on the RSF-selected features to further refine the gene signature and estimate the final model coefficients. The *α* parameter was fixed at 0.1 to balance L1 (LASSO) and L2 (ridge) regularization, favoring a slightly sparser model while maintaining grouping stability for correlated predictors. The optimal lambda was determined through 10-fold cross-validation in the training set using the minimum mean cross-validated error criterion. Genes with nonzero coefficients at the optimal lambda were included in the final risk model. The risk score for each patient was calculated using the following formula: Risk Score = Σ (*β_i_* × *Expr_i*), where *β_i_* represents the Enet coefficient for the *i*th selected gene and *Expr_i* denotes its *Z*-score-standardized expression value (standardized using the mean and standard deviation calculated from the training cohort).

### Evaluation of the lactylation-related gene model

Patients in the TCGA training set and the GSE29609 and E-MTAB-1980 validation sets were stratified into high- and low-risk groups based on their median risk scores derived from the lactylation-related prognostic model. Kaplan–Meier survival curves were generated using the survminer R package. Statistical significance was assessed using the log-rank test. Subgroup analyses were performed to evaluate survival differences across TNM stage categories. Receiver operating characteristic (ROC) curves were generated using the timeROC R package to evaluate the performance of the lactylation-related prognostic model in predicting overall survival (OS). Spearman correlation analysis was used to examine the correlation between the lactylation-related risk score and clinical variables, including age, sex, T stage, N stage, and M stage. Univariate and multivariate Cox regression analyses were performed in the TCGA-KIRC cohort to confirm the lactylation-related prognostic model as an independent prognostic factor. Finally, a nomogram integrating the lactylation-related risk score with key clinical parameters (age and TNM stage) was constructed to predict 1-, 3-, and 5-year survival probabilities. The discriminative accuracy and calibration of the nomogram were assessed using the concordance index and calibration curves, respectively. The net clinical benefit of the nomogram was evaluated using decision curve analysis.

### Tumor mutational burden analysis

Intratumor heterogeneity (ITH) represents the genetic and phenotypic diversity among cancer cells within individual tumors. Genetic ITH was quantified using the mutant-allele tumor heterogeneity (MATH) score derived from whole-exome sequencing data, which measures the dispersion of somatic variant allele frequencies. Somatic mutations in TCGA-KIRC were visualized using the R package “maftools.”

### Comprehensive analysis of immune characteristics in various risk subgroups

We further evaluated the association between the lactylation-related gene model and immune infiltration in ccRCC by first applying the CIBERSORT algorithm to quantify the proportions of 22 immune cell subsets. The results were visualized as violin plots across various risk-stratified groups. To reinforce the reliability of these findings, the ESTIMATE and ssGSEA algorithms were integrated to systematically examine the correlation between the lactylation-related gene model and the tumor immune microenvironment (TIME). The IMvigor 210 cohort was used to validate the predictive utility of the lactylation-related gene model for assessing immunotherapeutic responsiveness. Then, the model scores were compared among distinct therapeutic response categories (progressive disease, stable disease, partial response, and complete response).

### Cell lines and cell culture

The normal human renal tubular epithelial cell line (HK2) and five renal carcinoma cell lines (ACHN, 786-O, OS-RC2, Caki-2, and A498) were obtained from the Department of Urology, Shanghai East Hospital. All cell lines were routinely tested for mycoplasma contamination every 2 weeks during culture to exclude contamination. HK2, ACHN, and A498 cells were maintained in DMEM supplemented with 10% FBS, whereas 786-O, OS-RC-2, and Caki-2 cells were cultured in RPMI-1640 medium containing 10% FBS.

### Cell transfection

HEK293T cells were co-transfected with the specified lentiviral vector, the psPAX2 packaging plasmid, and the pMD2.G envelope plasmid. We designed and synthesized the corresponding short hairpin RNA (shRNA) sequences to achieve stable transfection and knockdown of *CDT1* expression: 5′-GCAGCUGUUGUACUAUCAU-3′ and 5′-GACUCGUGCUGCCCUACAA-3′. Subsequently, target cells (786-O and OS-RC2) were incubated with the resulting lentivirus and polybrene (at a final concentration of 1 µg/mL) for 24 h. After an additional 48 h of culture, the transduction efficiency was evaluated using real-time polymerase chain reaction (RT-PCR) and western blot analysis.

### Real-time polymerase chain reaction

First, total RNA was extracted from different cell types primarily using a commercial RNA extraction kit (R4111-02; Magen, Shanghai, China). The extracted total RNA was reverse transcribed using the HiScript II One-Step RT-PCR Kit (P611-01, Vazyme, Nanjing, China). RT-PCR analysis was performed using ChamQ SYBR RT-PCR Master Mix (Q341-02, Vazyme) for mRNA detection. The gene-specific primer sequences used in the study were as follows: CDT1, forward primer 5’-GGAGGTCAGATTACCAGCTCAC-3’ and reverse primer 5’-TTGACGTGCTCCACCAGCTTCT-3’; β-actin, forward primer 5’-AAGGTGACAGCAGTCGGTT-3’ and reverse primer 5’-TGTGTGGACTTGGGAGAGG-3’. Finally, β-actin-normalized cycle threshold values were analyzed using the 2–^ΔΔCT^ method.

### Western blot analysis

Total proteins were extracted using RIPA lysis buffer supplemented with 1× protease inhibitor mixture and 1× phosphatase inhibitor mixture. Protein concentration was quantified using the BCA protein assay kit (Catalog No. ZJ101, Epizyme, Shanghai, China). Proteins were separated by sodium dodecyl sulfate–polyacrylamide gel electrophoresis (SDS-PAGE) on a 10% gel. The housekeeping antibody GAPDH (Catalog No. 60004-1-Ig, Wuhan, China) and anti-CDT1 antibody used in the experiment were purchased from Proteintech (Catalog No. 14382-1-AP, Wuhan, China).

### Detection of cell proliferation

To assess *in vitro* cell proliferation, a CCK-8 assay was performed. Specifically, 786-O or OS-RC2 cells were seeded at a density of 1000 cells per well in a 96-well plate. CCK-8 reagent was added to the cell suspension at a 1:10 ratio and incubated at 37°C for 2 h. At the designated time point, the absorbance was measured at 450 nm using a microplate reader (BioTek, USA). For determining colony formation capacity, 786-O and OS-RC2 cells were seeded at a density of 500 cells per well in six-well plates and cultured for approximately 10 days. Formed colonies were fixed with 4% paraformaldehyde for 10 min, stained with crystal violet, and counted. Additionally, treated 786-O and OS-RC2 cells were labeled using the EdU (Catalog No. C0075S, Beyotime, Shanghai, China) staining method, and cell proliferation was observed and recorded using a fluorescence microscope (Nikon, Japan).

### Detection of cell migration and invasion

To assess the effect of CDT1 on the migratory capacity of ccRCC cells, tumor cells were seeded in six-well plates. When cells reached 80% confluence, the monolayer was scraped off using a 200-μL pipette tip. At the same location, the images of migrating cells were captured using a microscope (Nikon, Japan) at 0 and 24 h.

Transwell assay was performed using a 24-well microporous chamber equipped with an 8-µm pore membrane (Corning Inc., NY, USA). The cells were cultured for 24 h in serum-free medium in the upper chamber, whereas the lower chamber contained medium supplemented with 20% FBS. Following fixation with 4% paraformaldehyde, the cells in the lower chamber were stained with crystal violet for 10 min. The chamber was rinsed with water to remove upper-layer cells, dried at room temperature, and photographed.

### Determination of L-lactate levels

The 786-O and OS-RC2 cells were seeded in six-well plates. L-lactate levels in the cells were measured using a lactate detection kit (Catalog No. S0208S, Beyotime, Shanghai, China) following the manufacturer’s protocol.

### Animal models

Male BALB/c nude mice (aged 4 weeks) were purchased from Beijing Vital River Laboratory Animal Technology (Beijing, China) and subcutaneously injected with OS-RC-2 (5e6 cells) in the left axilla. The mice exhibited measurable tumor growth after 4 weeks. After reaching the end of the experiment, the mice were euthanized by cervical dislocation and the xenograft tumors were excised for subsequent experimental use.

### Statistical analysis

All statistical analyses were performed using R software (version 4.3.3) and GraphPad Prism (version 10.1.2). We conducted univariate and multivariate Cox regression analyses to evaluate the prognostic value and independence of the risk scores. The differences in risk scores and treatment response across subgroups within the TCGA-KIRC cohort were compared using the Wilcoxon signed-rank test. Experimental data were presented as mean ± standard deviation. Comparisons between two groups were performed using the *t* test, whereas comparisons among multiple groups were conducted using one-way analysis of variance. A *P* value less than 0.05 indicated a statistically significant difference.

## Results

### Single-cell sequencing analysis and gene set scoring

**Figure 1:**
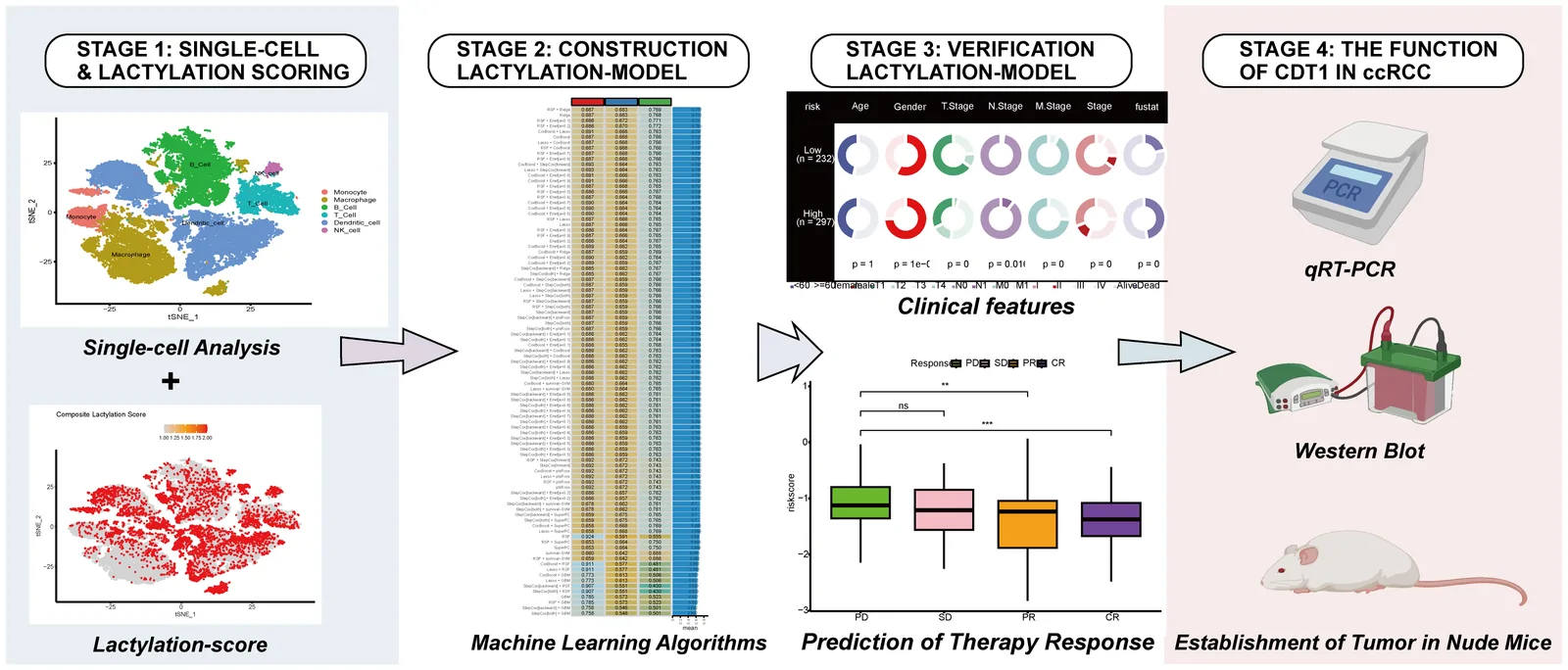
Flowchart of the study design.

**Figure 2:**
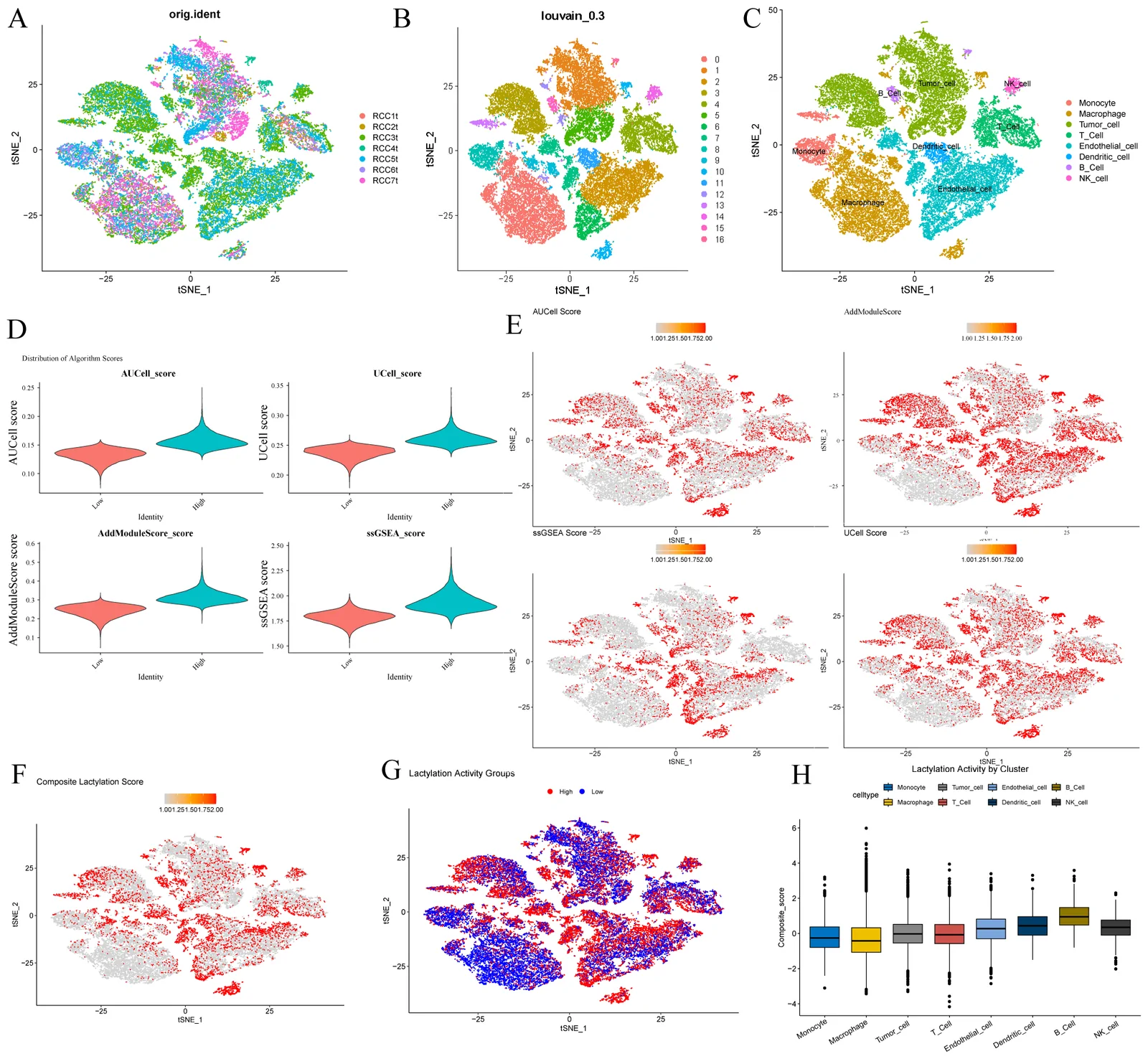
Clustering, annotation, and lactylation scoring of single-cell datasets for ccRCC. **(a)** t-SNE visualization of cell distribution across seven ccRCC samples. **(b)** Cell clusters mapped in t-SNE space. **(c)** Annotation of cell subpopulations overlaid on t-SNE coordinates. **(d)** Violin plots and **(e)** t-SNE heatmaps showing scores derived from four single-cell scoring algorithms. **(f)** Composite score distribution across single-cell subpopulations visualized on t-SNE heatmaps. **(g)** Score grouping (high vs low) depicted on t-SNE heatmaps of single-cell subpopulations. **(h)** Box plots of lactylation-related score for distinct cell subpopulations.

The overall workflow of this study was illustrated in **Figure 1**. The single-cell dataset GSE156632 included seven ccRCC samples (**Figure 2a**). After batch effect correction using the Harmony R package, the cells were relatively uniformly distributed across samples. Using gene expression profiles, we identified 17 clusters (**Figure 2b**), annotated them using cell-specific markers, and classified them into 8 cell types: tumor cells, monocytes, macrophages, T cells, B cells, dendritic cells, endothelial cells, and natural killer cells (**Figure 2c**). Applying four single-cell scoring algorithms, we evaluated the transcriptional enrichment of lactylation-associated gene sets (**Figure 2d**). The distributions of scores across cell subpopulations for all four algorithms are shown in **Figure 2e**. We integrated the scores from the four algorithms to minimize algorithmic bias and visualized the composite results. The cells were stratified into high- and low-score groups based on the median composite score (**Figures 2f, g**). The box plots revealed that lactylation-related gene expression signatures were detected across multiple cell types in the ccRCC microenvironment, with B cells displaying relatively higher scores (**Figure 2h**).

### Identification of key modules and genes using WGCNA and survival analyses

**Figure 3:**
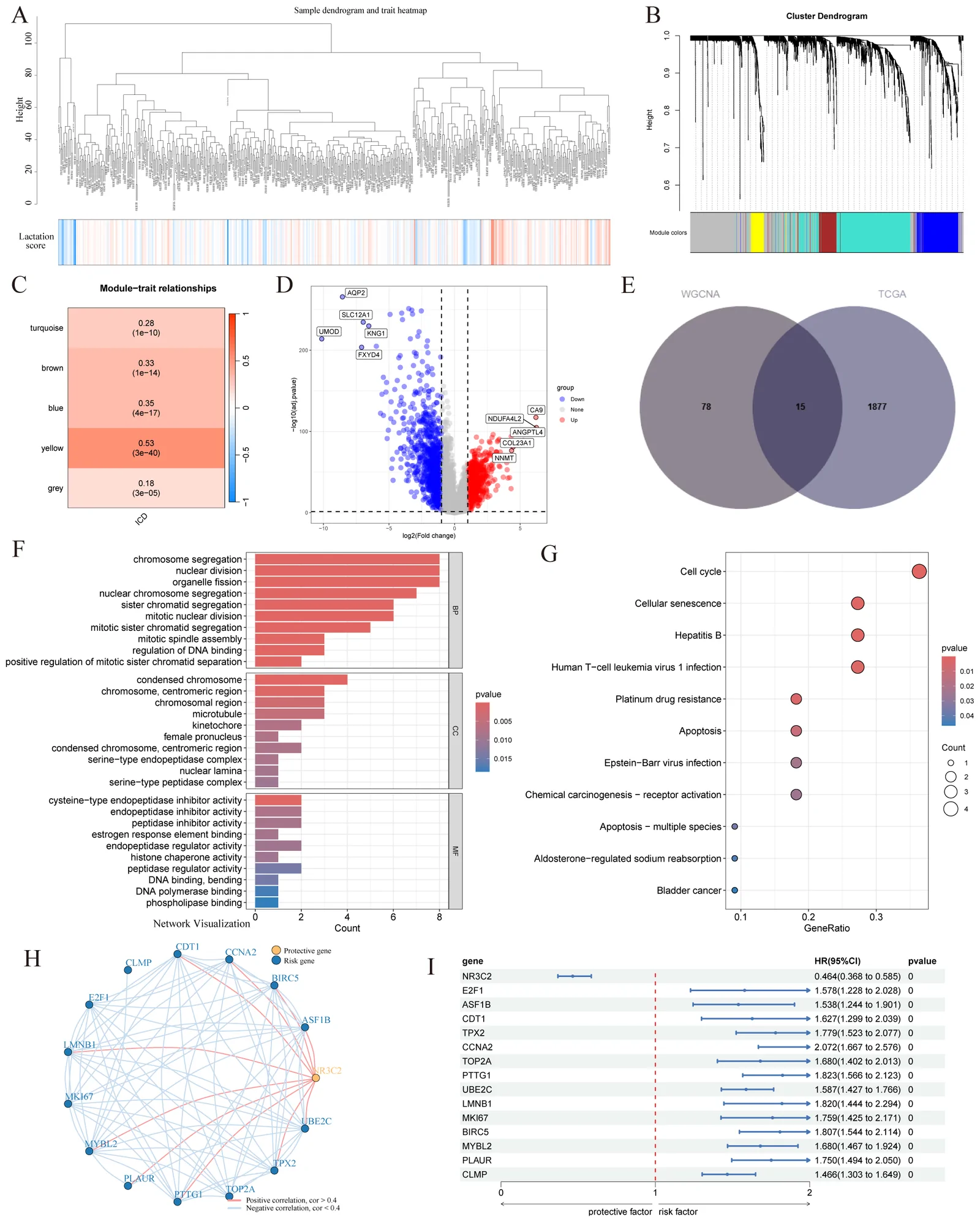
Identification of lactylation-associated features in single-cell transcriptomes of ccRCC and lactylation-related genes via WGCNA. **(a)** Hierarchical clustering of ccRCC samples. **(b)** Clustering tree from WGCNA analysis. **(c)** Heatmap of module - lactylation score associations. **(d)** Volcano plot of differentially expressed genes in ccRCC versus normal tissues. **(e)** Venn diagram of overlapping genes identified by WGCNA and TCGA differential expression analysis. **(f, g)** Functional enrichment analysis of the 15 common genes: **(f)** GO terms and **(g)** KEGG pathways. **(h)** Interaction network of prognostic lactylation_related genes. **(i)** Forest plot showing Cox regression results for lactylation-related genes and overall survival.

We obtained lactylation-related transcriptional scores for each TCGA-KIRC sample using the composite algorithm as input for WGCNA. To identify modules and genes associated with these scores, we first removed outlier samples and then constructed a co-expression network for the 2050 lactylation-related genes identified from single-cell transcriptomic analysis (**Figure 3a**; **Supplementary Table 2**). The optimal soft-thresholding power was set to 10 and the minimum module size to 50 genes, yielding 5 modules (**Figure 3b**). Subsequent analysis revealed that the MEYellow module was strongly correlated with lactylation-related transcriptional scores (**Figure 3c**). This module contained 93 genes (**Supplementary Table 3**).

We identified 1892 DEGs from TCGA using thresholds of |logFC| >1 and adjusted *P* < 0.05 (**Figure 3d**). The intersection of these DEGs with the 93 genes in the MEYellow module yielded 15 overlapping genes (**Figure 3e**). These genes were consistently associated with lactylation-related transcriptional patterns in ccRCC across both bulk and single-cell transcriptomic contexts. Functional enrichment analysis revealed significant enrichment in biological processes including chromosome segregation and nuclear division, cellular components including condensed chromosomes and centromeric regions, and molecular functions such as cysteine-type endopeptidase inhibitor activity and endopeptidase inhibitor activity (**Figure 3f**). KEGG pathway analysis indicated significant enrichment in cell cycle and cellular senescence pathways (**Figure 3g**). Cox regression analysis revealed that all 15 overlapping genes (*NR3C2*, *E2F1*, *ASF1B*, *CDT1*, *TPX2*, *CCNA2*, *TOP2A*, *PTTG1*, *UBE2C*, *LMNB1*, *MKI67*, *BIRC5*, *MYBL2*, *PLAUR*, and *CLMP*) were significantly associated with ccRCC prognosis (all *P* < 0.05) (**Figures 3h, i**).

### Construction of a lactylation-related prognostic model using machine learning algorithms

**Figure 4:**
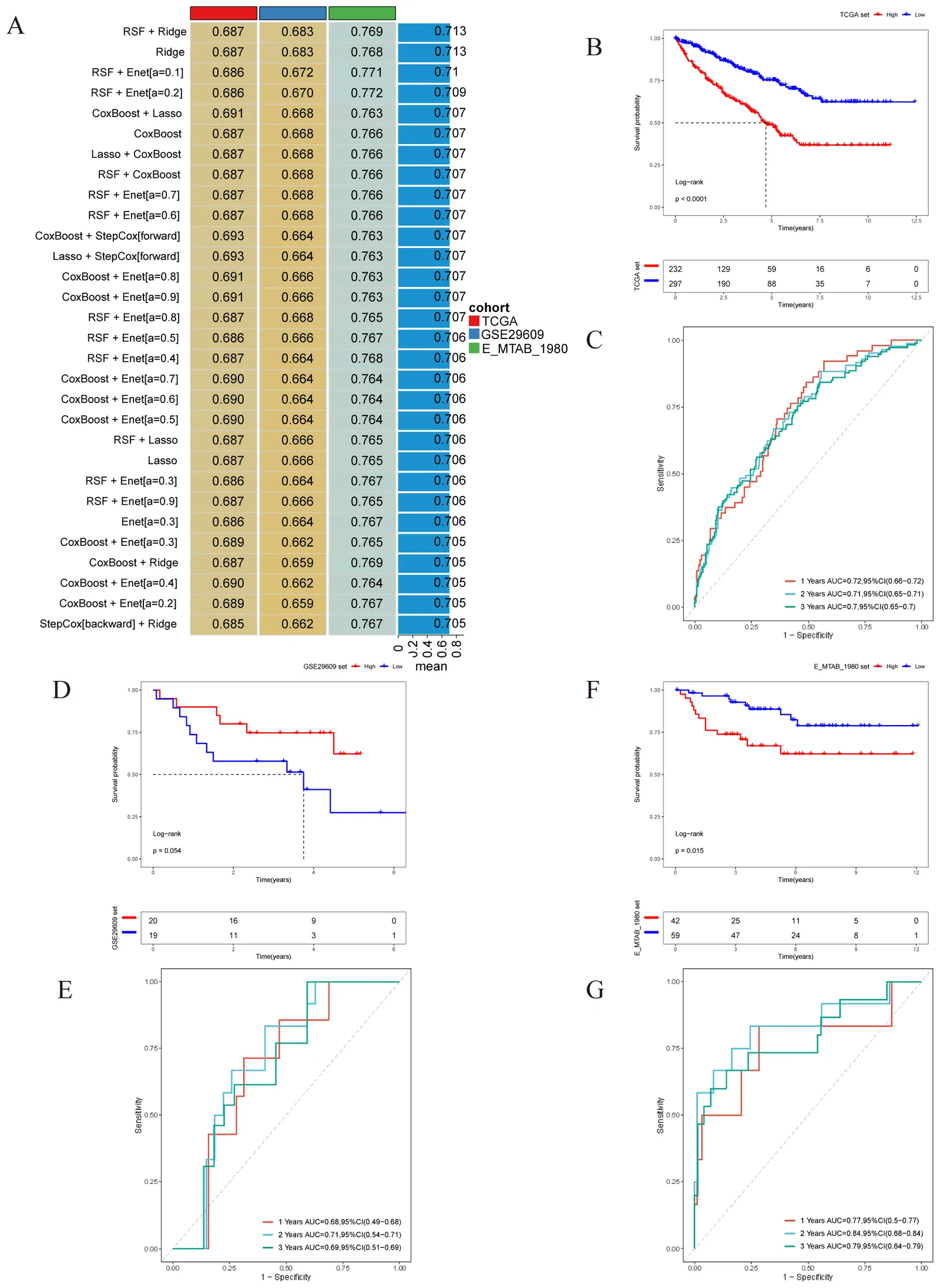
Developing and validating optimal lactylation-related ccRCC prognostic models using machine learning algorithms. **(a)** Construction of 101 prediction models with further validation across two independent validation sets. The top 30 models are shown. Kaplan–Meier survival curves of OS according to lactylation-related gene model characteristics across three datasets: **(b)** TCGA, **(d)** GSE29609, and **(f)** E-MTAB-1980. The lactylation-related prognostic model predictive performance: ROC for 1/2/3-year OS in **(c)** TCGA, **(e)** GSE29609, and **(g)** E-MTAB-1980.

In this study, TCGA-KIRC served as the training dataset whereas the GSE29609 and E-MTAB-1980 cohorts were used as external validation datasets. We constructed prediction models using 10 machine learning algorithms and their 101 permutations, and evaluated their performance by calculating consistency across each validation dataset. Among all candidate models, LASSO achieved a C-index of 0.706 (training set: 0.687; validation set: 0.666–0.765), ridge achieved a C-index of 0.713 (training set: 0.687; validation set: 0.683–0.768), and RSF + ridge achieved a C-index of 0.713 (training set: 0.687; validation set: 0.683–0.769). Although the overall C-index values were comparable, RSF + Enet (*α* = 0.1) exhibited better accuracy in one independent validation set (0.771) and the most balanced performance across all cohorts (C-index: 0.710; training set: 0.686; validation set: 0.672–0.771). We randomly selected 50% of the patients from the TCGA cohort as an internal validation set and found that the C-index remained stable at 0.677. Therefore, RSF + Enet (*α*  =  0.1) was selected as the final prognostic model. Based on evaluations in both training and validation sets, we selected the RSF + Enet (*α* = 0.1) algorithm to build a prediction model using 13 hub genes (*NR3C2*, *E2F1*, *ASF1B*, *CDT1*, *TPX2*, *CCNA2*, *TOP2A*, *PTTG1*, *LMNB1*, *MKI67*, *BIRC5*, *MYBL2*, and *PLAUR*) (**Figure 4a**).

We assigned all patients to either the high- or low-risk group based on their median risk scores. Notably, in the TCGA-KIRC training set, patients in the high-risk group demonstrated significantly poor OS compared with those in the low-risk group (**Figure 4b**). Similarly, the validation set E-MTAB-1980 revealed worse OS in the high-risk group (**Figure 4d**). However, the validation set GSE29609 displayed inconsistent survival trends, though without statistical significance (**Figure 4f**). We hypothesized that this discrepancy might have stemmed from insufficient sample size (*N* = 39). ROC curve analysis revealed that, in the TCGA-KIRC dataset, the area under the curve (AUC) for the lactylation model reached 0.72, 0.71, and 0.70 at 1-year, 2-year, and 3-year intervals, respectively (**Figure 4c**). In GSE29609, the AUC values were 0.68, 0.71, and 0.69, respectively (**Figure 4e**). In the E-MTAB-1980 dataset, the AUC values were 0.72, 0.71, and 0.70, respectively (**Figure 4g**). Regarding prognostic prediction for ccRCC, an AUC of approximately 0.7 in independent validation cohorts indicated moderately accurate and clinically meaningful performance, which was comparable to many established gene signatures in the field.

### Clinical feature prognostic analysis of lactylation-related gene model

**Figure 5:**
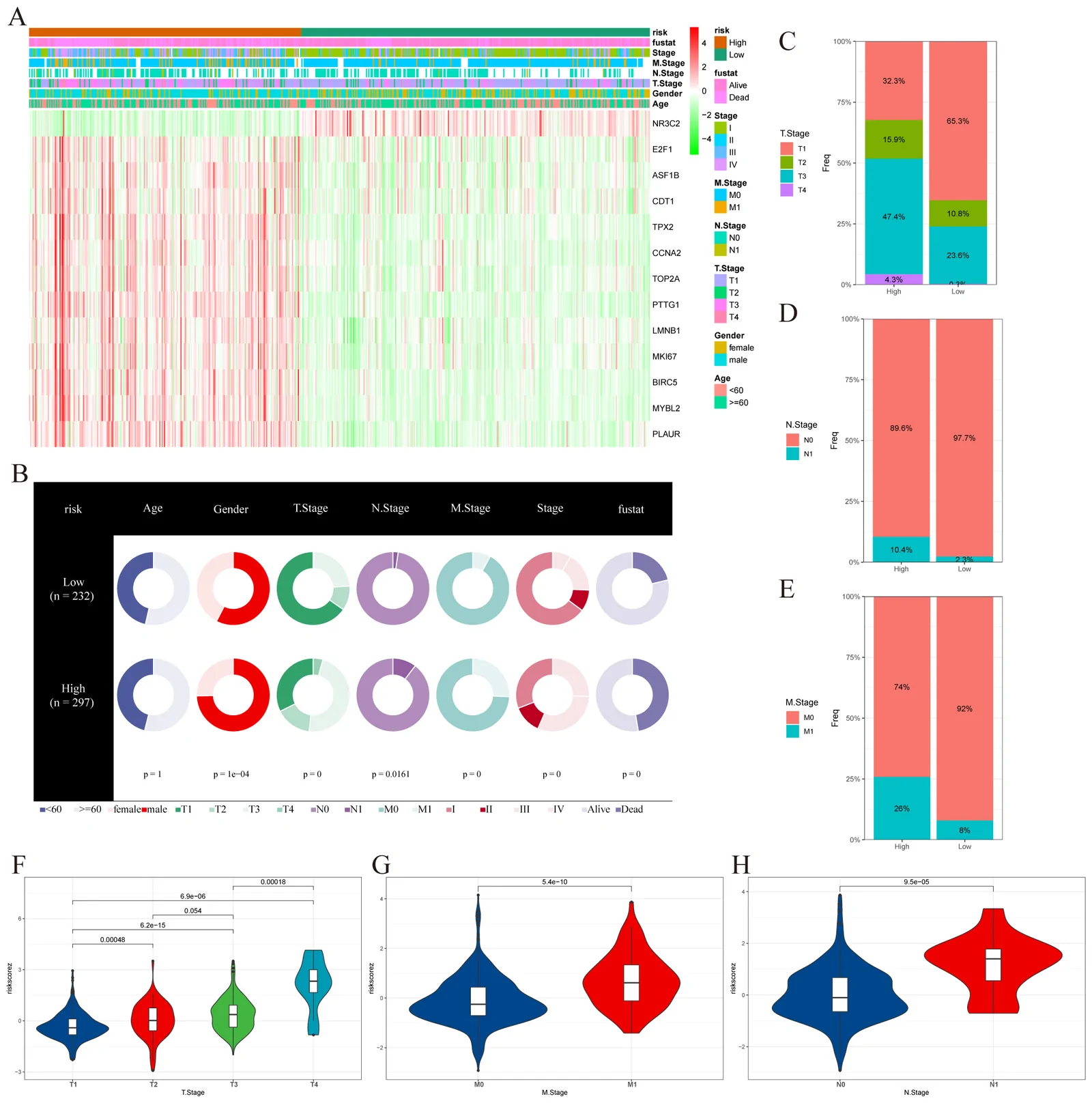
Evaluation of the lactylation-related prognostic model in ccRCC. **(a)** Clinical characteristics and expression profiles of key genes across risk-score groups. **(b)** Association between the lactylation-related prognostic model risk groups (low/high) and clinical features. **(c–e)** Distribution of **(c)** T stage, **(d)** N stage, and **(e)** M stage in lactylation-model risk subgroups. **(f–h)** Risk-score comparisons across patient groups stratified by **(f)** T stage, **(g)** M stage, and **(h)** N stage.

We evaluated the correlation between lactylation and various clinical characteristics. We observed significant differences in the TCGA-KIRC dataset (*P* < 0.05) in terms of pathological staging, T stage, N stage, M stage, and sex distribution between the high- and low-risk groups (**Figures 5a, b**). The stacked bar chart indicated higher proportions of T3-4, N1, and M1 in the high-risk group (**Figures 5c–e**). The violin plot revealed elevated risk scores across the T4, N1, and M1 subgroups (**Figures 5f–h**).

We performed univariate and multivariate Cox regression analyses of OS in the TCGA-KIRC dataset to evaluate whether the lactylation-related gene model was an independent prognostic factor for ccRCC (**Supplementary Figures 1a, b**). The univariate analysis indicated the lactylation-related gene model as a significant risk factor for OS (HR = 1.864, 95% CI: 1.636–2.124, *P* < 0.001). The multivariate analysis revealed that the lactylation model remained an independent prognostic factor influencing OS (HR = 1.685, 95% CI: 1.349–2.105, *P* < 0.001). We constructed a nomogram integrating lactylation-related gene model data with clinical characteristics to enhance the clinical applicability of the lactylation-related gene model (**Supplementary Figure 1e**). The calibration curves demonstrated excellent agreement between nomogram predictions and actual observations (**Supplementary Figure 1c**). The decision curve analysis confirmed that the nomogram yielded superior net clinical benefit compared with other clinical features (**Supplementary Figure 1d**). We performed functional enrichment analysis to further investigate the molecular mechanisms linking the lactylation-related gene model to ccRCC prognosis. GSEA based on GO gene sets showed that the high-risk group was enriched in the TNF-α signaling via NF-κB pathway and the IL-6–JAK–STAT3 signaling pathway, whereas the low-risk group was enriched for pathways associated with fatty acid metabolism and adipogenesis regulation (**Supplementary Figures 1f, g**).

### Molecular mechanisms and tumor mutation burden in the bulk transcriptome

As shown in **Supplementary Figure 2a**, patients with ccRCC in the high-risk group had higher MATH scores than those in the low-risk group, despite no statistically significant difference. We further investigated the relationship between MATH and prognosis in patients with ccRCC. Patients with high MATH scores had poorer OS than those with low MATH scores, but this difference was not statistically significant (log-rank *P* = 0.35) (**Supplementary Figure 2b**). The combined stratification by risk group and MATH score revealed that the high-risk/high-MATH group had the worst OS (log-rank *P* < 0.001) (**Supplementary Figure 2c**). We mapped the mutation landscape of the high- and low-risk groups to investigate genomic differences between subgroups of the lactylation-related model (**Supplementary Figures 2d, e**). The analysis of co-occurring and mutually exclusive mutations among the top 20 mutated genes showed that co-occurring mutations were more frequent in the high-risk group (**Supplementary Figures 2f, g**).

### Relation of lactylation-related gene model with single-cell characteristics and immune features

**Figure 6:**
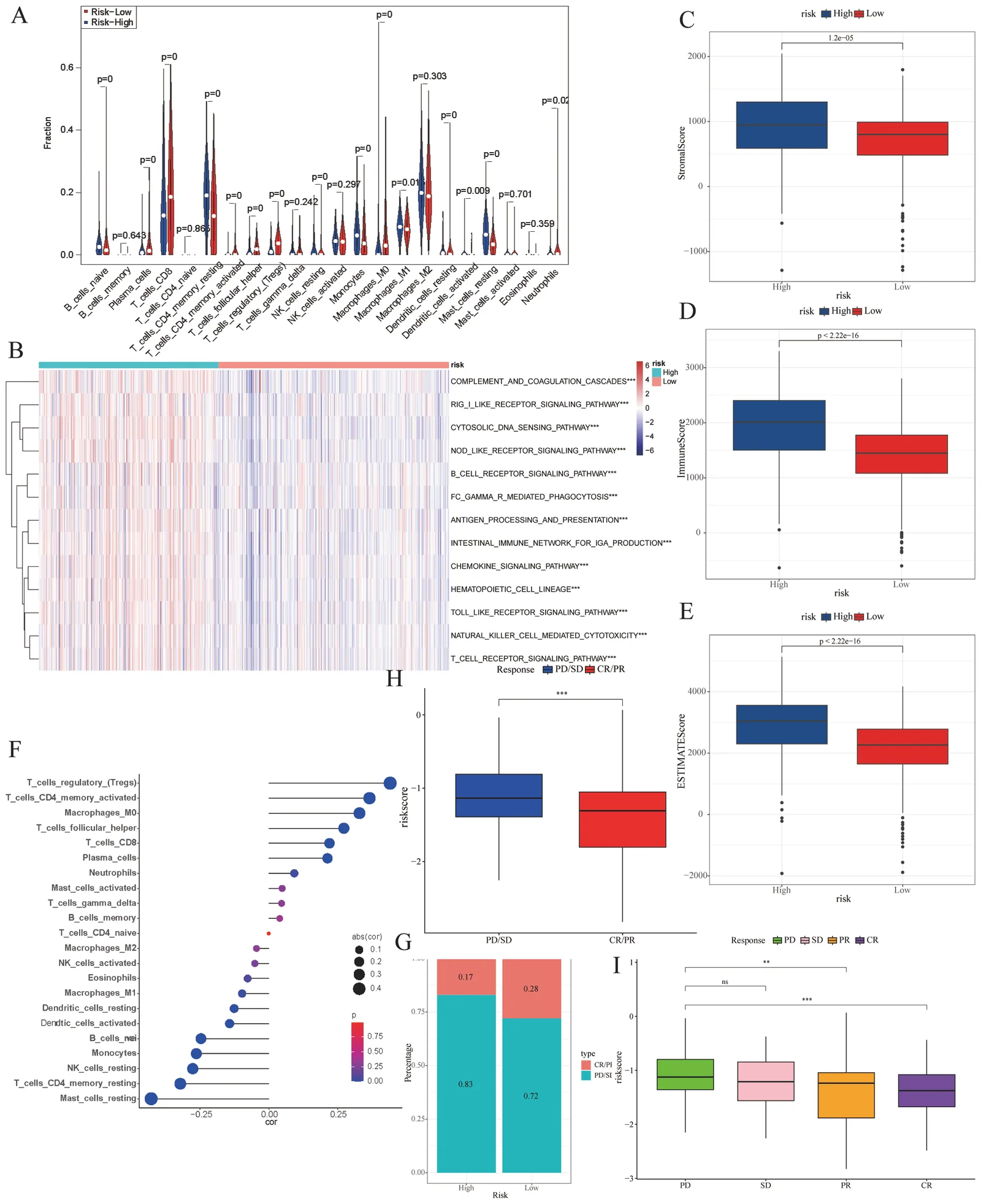
Relationship between the lactylation-related prognostic model and the immune landscape in ccRCC. **(a)** Quantification of the abundance of each immune cell type between high- and low-risk groups according to the CIBERSORT algorithm. **(b)** Immune pathway activity of different risk groups. **(c–e)** Immune microenvironment metrics by risk group: **(c)** stromal score, **(d)** immune score, and **(e)** ESTIMATE score. **(f)** Relationship between TME-infiltrating cells and the lactylation-related gene model of ccRCC. **(g)** Risk score distribution for different treatment outcomes in the IMvigor210 cohort. **(h, i)** Box plots showing **(h)** risk scores and **(i)** differences in risk scores among different subgroups (CR, PR, SD, and PD). *p < 0.05, **p < 0.01, ***p < 0.001; and ns, not significant.

We first analyzed the expression of 13 hub genes in various immune cells at the single-cell transcriptomic level (**Supplementary Figure 3**). Then, we classified patients in the TCGA-KIRC cohort into high- and low-risk groups using the lactylation-related gene model score. The abundance of infiltrating immune cells in each sample was quantified using the CIBERSORT algorithm to analyze the differences in specific immune cell infiltration between the two groups. The study revealed that the proportions of both CD8+ T cells and activated CD4+ T cells were significantly elevated whereas the proportions of M1 macrophages were significantly reduced in the low-risk group (**Figure 6a**). We obtained immune-related pathway scores by applying the ssGSEA algorithm, revealing significantly higher activity of the complement and coagulation cascades and the B cell receptor signaling pathway in the high-risk group (**Figure 6b**). We also applied the ESTIMATE algorithm to calculate the immune score, stromal score, ESTIMATE score, and tumor purity score for the lactylation-related gene model risk subgroups. The high-risk group demonstrated significantly higher tumor purity scores, immune scores, and ESTIMATE scores (**Figures 6c–e**). Next, we used Spearman’s correlation analysis to identify immune cell types significantly associated with the lactylation model, with Treg cells exhibiting the strongest correlation (**Figure 6f**).

The prediction of response to immunotherapy revealed a significantly lower proportion of complete remission/partial remission (CR/PR) in the high-risk group (**Figure 6g**). Furthermore, patients with CR/PR had significantly lower risk scores than those with SD/PD (**Figures 6h, i**).

### Expression of *CDT1* in ccRCC

**Figure 7:**
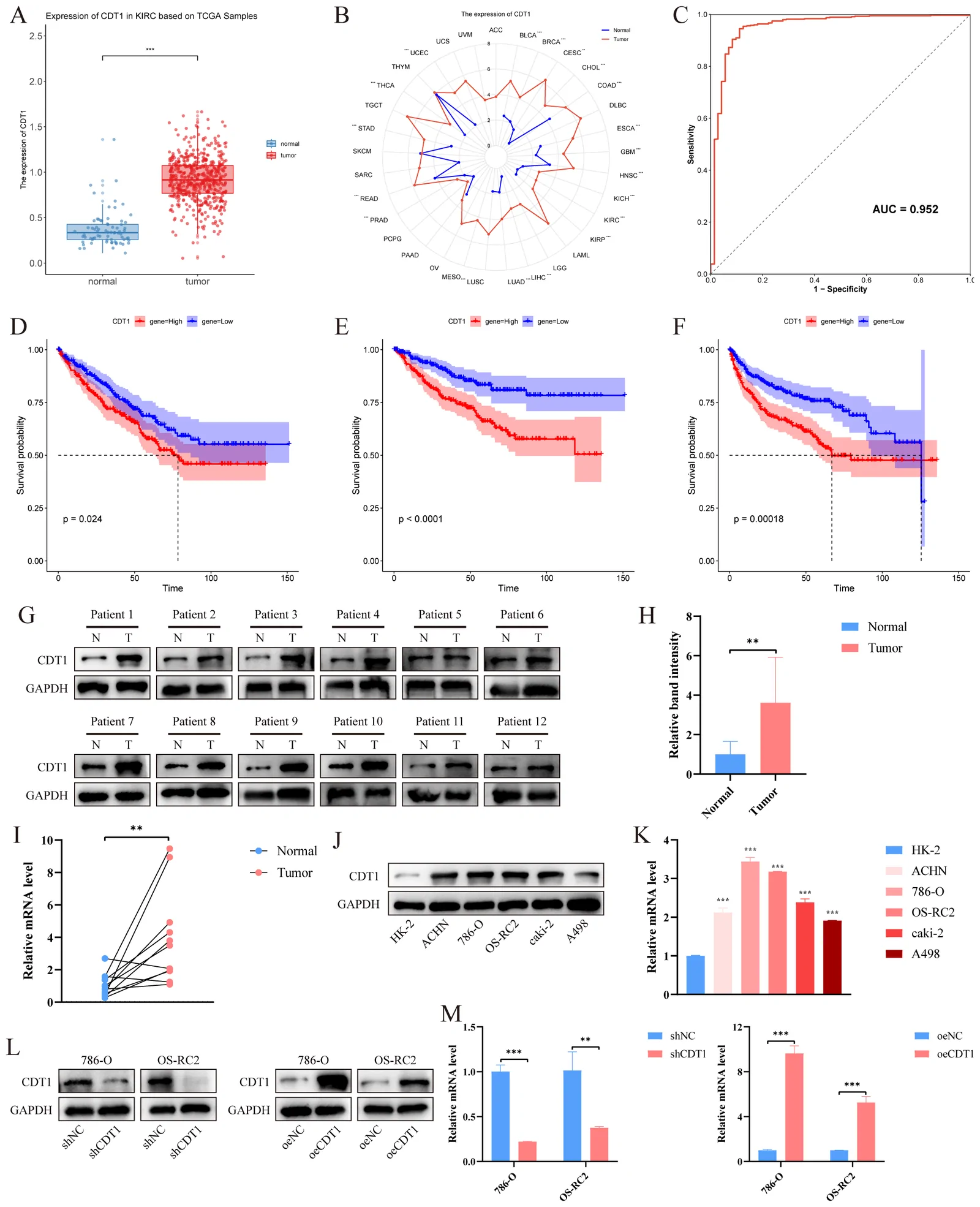
*CDT1* expression in ccRCC and validation of its knockdown and overexpression in two cell lines (786-O and OS-RC2). **(a)** *CDT1* expression in ccRCC (TCGA data). **(b)** Pan-cancer landscape of *CDT1* expression. **(c)** Diagnostic ROC curve for *CDT1* in ccRCC. **(d-f)** Kaplan–Meier survival analysis based on *CDT1* expression in ccRCC: **(d)** overall survival (OS), **(e)** disease-specific survival (DSS), and **(f)** progression-free survival (PFS). **(g, h)** CDT1 protein levels in ccRCC tumor versus adjacent nontumor tissues. **(i)** Relative mRNA levels of CDT1 in ccRCC tumor versus adjacent nontumor tissues. **(j, k)** CDT1 **(j)** protein and **(k)** mRNA levels across ccRCC cell lines. **(l, m)** *CDT1* expression was significantly downregulated in CDT1-KD cells and upregulated in oeCDT1 cells. *p < 0.05, **p < 0.01, ***p < 0.001; and ns, not significant.

According to TCGA-KIRC data, *CDT1* was significantly overexpressed in tumors (**Figure 7a**). Pan-cancer analysis further revealed that *CDT1* exhibited high expression across multiple tumor types, including bladder cancer, prostate cancer, gastric cancer, and colorectal cancer (**Figure 7b**). Notably, *CDT1* demonstrated favorable diagnostic properties for ccRCC (**Figure 7c**). The survival analysis indicated that patients with high *CDT1* expression had poorer OS, disease-specific survival (DSS), and progression-free survival (PFS) (**Figures 7d–f**).

Western blot and RT-PCR analyses of tumor and adjacent noncancerous tissues from 12 patients revealed higher *CDT1* expression in tumor tissues (**Figures 7g–i**). The baseline clinical characteristics of these clinical and experimental samples are presented in **Supplementary Table 5**. Detection of *CDT1* expression in HK2 and five kidney cancer cells revealed higher expression in tumor cells, with relatively higher levels in 786-O and OS-RC2 cells, which were selected for subsequent experiments (**Figures 7j, k**). *CDT1* knockdown and overexpression models were established in these two cell lines, respectively, and the successful establishment of both cellular models was confirmed (**Figures 7l, m**).

### *In vitro* experiments validating the function of *CDT1* in ccRCC

**Figure 8:**
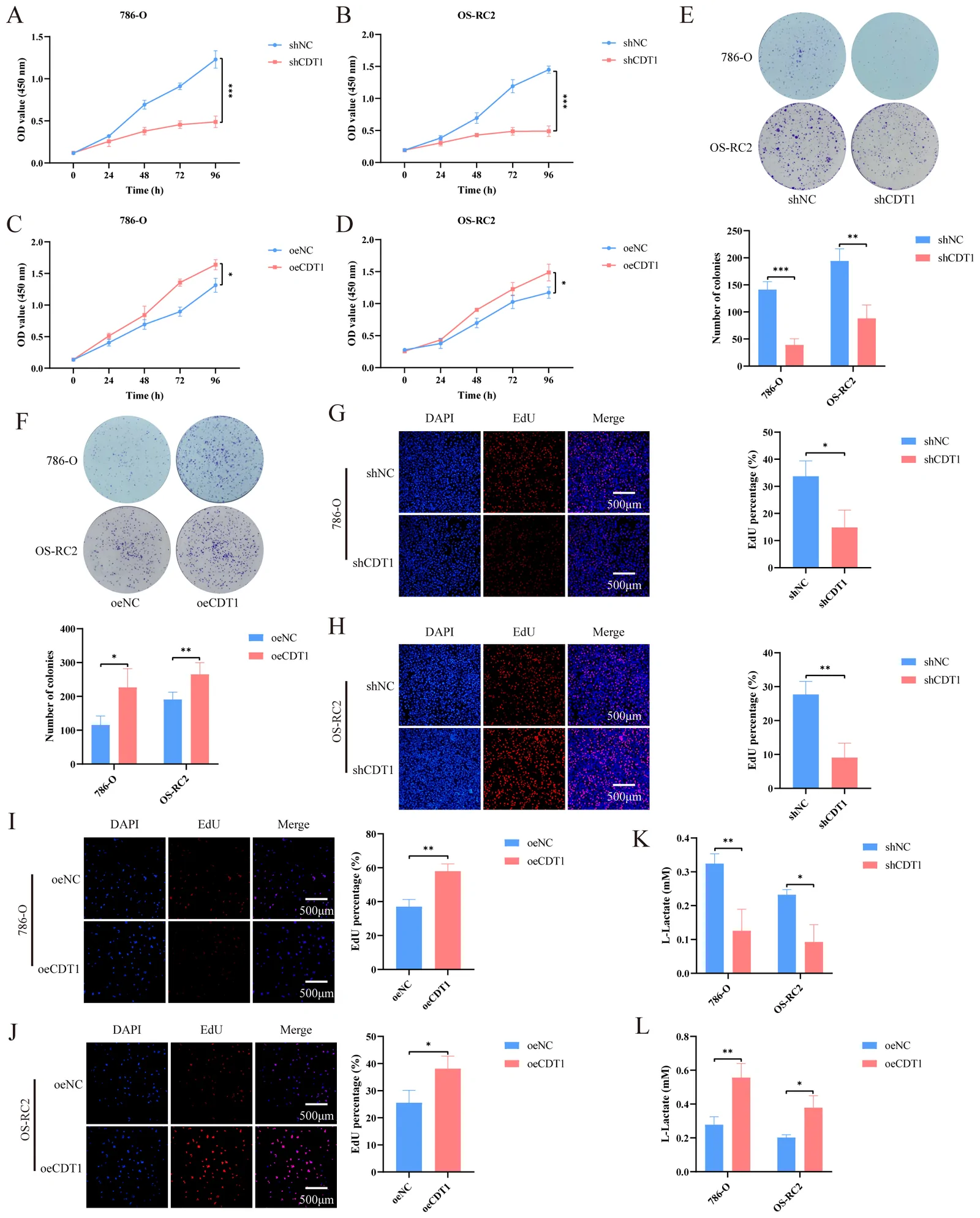
*CDT1* promoted proliferation of ccRCC cells. **(a–d)** CCK-8 analysis of the effect of *CDT1* knockdown or overexpression on ccRCC cell growth. **(e, f)** Colony formation assay investigating the effects of *CDT1* on ccRCC cell proliferation. **(g–j)** EdU assay for the effect of *CDT1* on ccRCC cell proliferation. **(k, l)** Quantification of intracellular L-lactate levels among different treatment groups. *p < 0.05, **p < 0.01, ***p < 0.001; and ns, not significant.

CCK-8 assay results indicated that sh-*CDT1* significantly suppressed the growth of both 786-O and OS-RC2 cells, whereas oe-*CDT1* markedly promoted the growth of these two cell lines (**Figures 8a–d**). The colony formation assay further corroborated these findings (**Figures 8e, f**). The EdU assay demonstrated that the proportion of cells in the proliferative phase was lower in the sh-*CDT1* group compared with the control group, whereas it was higher in the oe-*CDT1* group (**Figures 8g–j**). Detection of L-lactate in the cells revealed lower L-lactate levels in the sh-*CDT1* group and higher levels in the oe-*CDT1* group (**Figures 8k, l**). Notably, these changes in intracellular L-lactate might reflect secondary metabolic adaptations driven by altered proliferative activity, rather than a direct regulatory role of *CDT1* in lactate production or lactylation.

**Figure 9:**
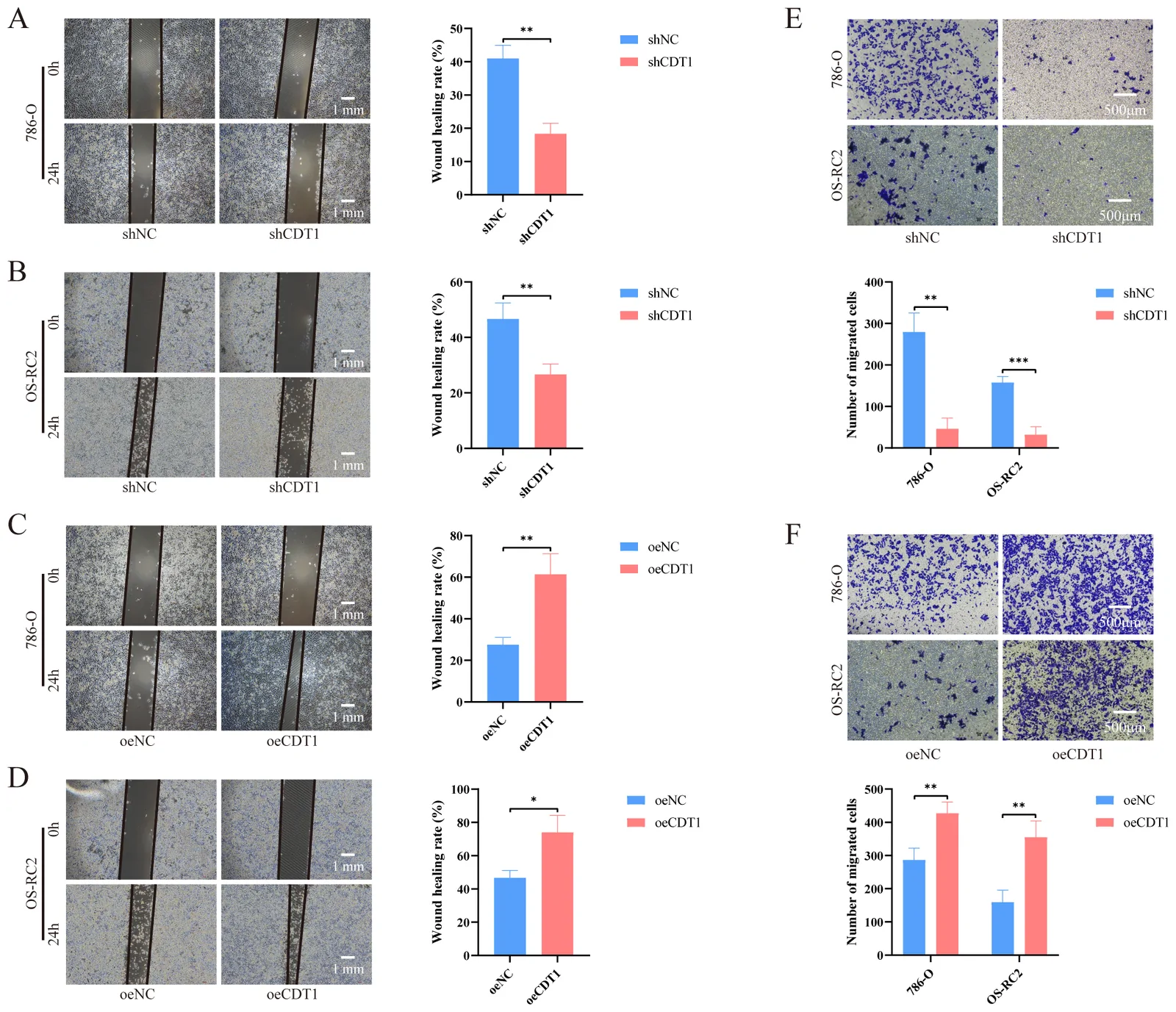
*CDT1* promoted migration and invasion of ccRCC cells. **(a–d)** Wound healing assay showing the effects of *CDT1* on ccRCC cell migration. **(e, f)** Transwell assay for the effect of *CDT1* on ccRCC cell invasion. *p < 0.05, **p < 0.01, ***p < 0.001; and ns, not significant.

Wound healing and Transwell assays revealed that *CDT1* knockdown significantly reduced the migration rate and invasive capacity of ccRCC cells (**Figures 9a, b, e**). On the contrary, *CDT1* overexpression markedly promoted the migration and invasion of ccRCC cells (**Figures 9c, d, f**).

### *In vivo* experiments validating the function of *CDT1* in ccRCC

**Figure 10:**
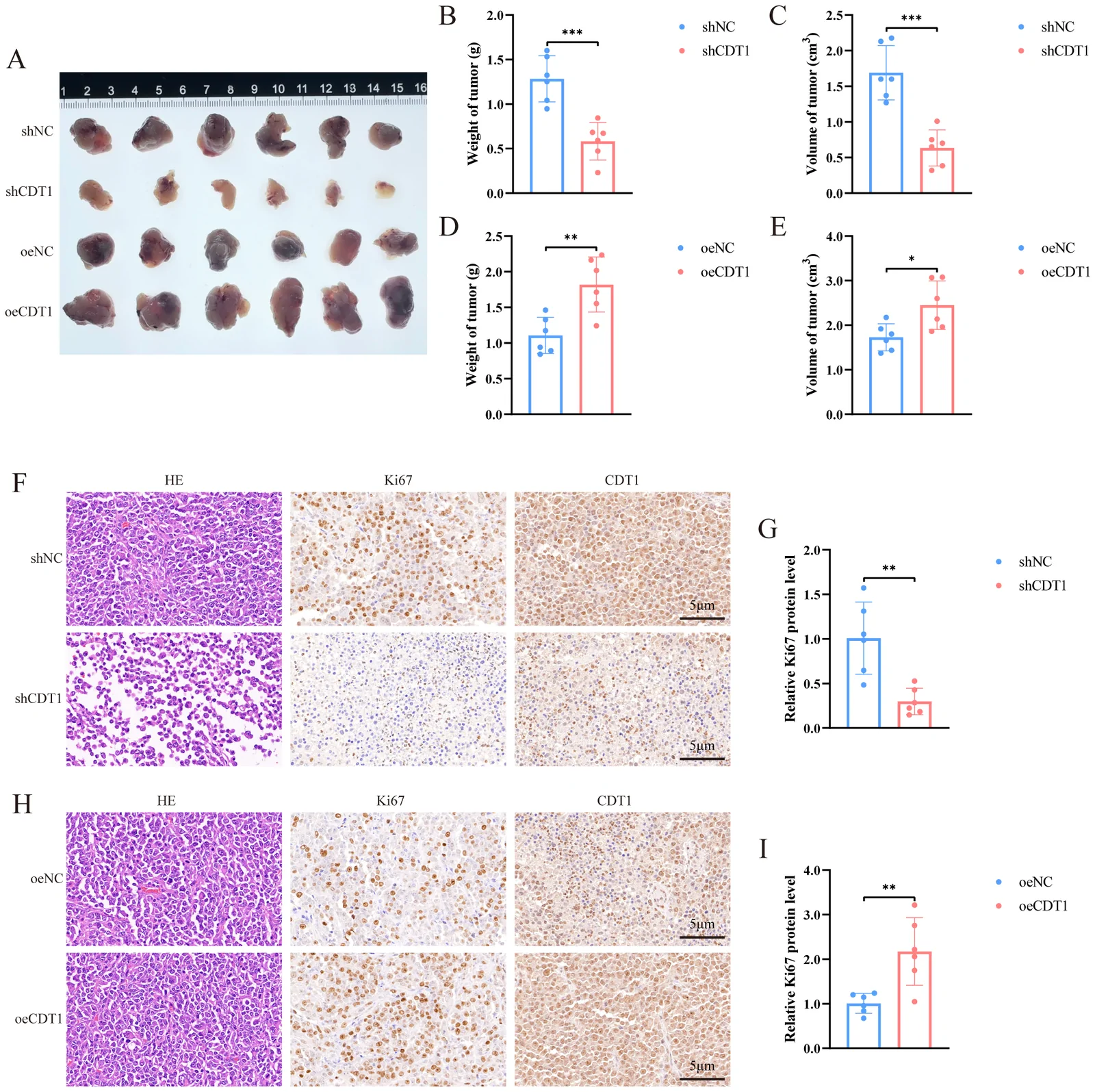
Role of *CDT1* in ccRCC in a nude mouse model. **(a)** Different groups of OS-RC-2 cells were injected subcutaneously into nude mice. **(b–e)** Tumor weight and tumor volume of different groups were statistically analyzed. **(f–i)** H&E staining and immunohistochemical staining to detect cell proliferation–related markers in mouse tumors. *p < 0.05, **p < 0.01, ***p < 0.001; and ns, not significant.

*In vivo* experiments demonstrated that tumors in the sh-*CDT1* group grew more slowly, whereas tumors in the oe-*CDT1* group grew more rapidly compared with those in the control group (**Figures 10a–e**). Ki-67 staining indicated that *CDT1* knockdown led to inhibition of cell proliferation. In contrast, *CDT1* overexpression caused a significant increase in cell proliferation (**Figures 10f–i**).

## Discussion

Emerging evidence indicates the involvement of lactylation in multiple pathological processes, including the regulation of the tumor microenvironment. In this study, we integrated single-cell and bulk transcriptomic data based on 364 lactylation-related genes and applied a combination of 4 computational algorithms (AUCell, UCell, AddModuleScore, and ssGSEA) to rigorously identify key lactylation-related genes in ccRCC. We further constructed a prognostic prediction model using WGCNA along with 10 machine learning algorithms and their 101 combinations, and validated its performance with an external validation cohort. Through *in vitro* and *in vivo* experiments, we provided the first functional evidence that the model’s core gene, *CDT1*, promoted proliferation and metastasis in ccRCC. These experiments established *CDT1* as an oncogenic driver in ccRCC, but did not demonstrate that its oncogenic function was mediated by or dependent on lactylation. Collectively, this study offers a computational framework for investigating potential lactylation-associated mechanisms in ccRCC.

WGCNA has gained widespread application in bioinformatics in recent years (17), primarily for identifying gene modules from high-throughput data. To some extent, it facilitates the precise identification of disease-associated gene sets. We identified genes associated with lactylation in ccRCC by intersecting the most crucial module genes from WGCNA with the DEGs between tumor and adjacent normal tissues in the TCGA dataset, followed by functional enrichment analysis. Both the cell cycle and apoptosis are key signaling pathways influencing ccRCC cells. Meng et al. (18) found that the enrichment of histone lactylation at promoters was crucial for maintaining gene expression involved in cell cycle and DNA repair pathways. In cardiovascular diseases, lactylation can exert effects in two ways: on the one hand, by modifying histones to coordinate inflammation resolution and cell cycle activation; on the other hand, by modifying nonhistones to regulate contractility and pathological remodeling (19). In renal diseases, histone modifications play a central role in regulating gene expression and cellular behavior (20).

Beyond influencing the cell cycle, lactylation has been demonstrated to significantly impact the apoptotic process (21–23). A recent study revealed that CASP3 lactylation promoted apoptosis resistance in acute lymphoblastic leukemia (21). Lactylation also serves as a major driver of cellular senescence, finely tuning gene expression programs across multiple processes. Histone lactylation and CoA levels significantly decline during cellular senescence but recover under hypoxic conditions due to increased glycolytic activity. Furthermore, modulation of enzymes crucial for histone lactylation reduces lactylation, thereby accelerating cellular senescence (18). SIRT6-mediated epigenetic repression of inflammatory responses has been shown to mitigate renal tubular injury in diabetic kidney disease, underscoring the interplay between metabolic sensors and epigenetic regulation in renal pathophysiology (24). Moreover, emerging evidence suggests that traditional Chinese medicine may exert therapeutic effects in diabetic kidney disease by modulating DNA methylation, offering novel insights into epigenetic-targeted interventions for renal metabolic disorders (25). The pathway associated with platinum-based drug resistance merits focused attention. Sun et al. (26) revealed significant upregulation of histone lactylation in platinum-resistant ovarian cancer, with prominent enrichment observed specifically at the H3K9 site. They proposed that modulating lactylation of histones and RAD51 could represent a novel therapeutic strategy to overcome such resistance. Although similar mechanisms have not yet been documented in ccRCC, this approach holds potential translational relevance for patients with platinum-resistant malignancies. Of note, the traditional pathway enrichment analysis failed to detect significant lactylation-related genes. This is likely attributed to the inherent limitations of bulk RNA-seq in reflecting cell-type-specific features, as well as the fact that lactylation is a PTM that cannot be fully captured at the transcriptional level.

Numerous studies (27, 28) have confirmed the reliability of employing 10 machine learning algorithms and their 101 combinations for model construction. However, our model had one limitation: The risk-stratified survival curves derived from the GSE29609 dataset did not demonstrate significantly poorer survival in the high-risk group, which was inconsistent with the survival predictions obtained from both the TCGA training and E-MTAB-1980 validation sets. We attributed this discrepancy primarily to the extremely small sample size of the GSE29609 cohort (*N* = 39), which introduced substantial statistical instability and increased the risk of sampling variability. Additionally, the GSE29609 dataset lacked detailed treatment information, precluding adjustment for this key confounding factor. Consequently, we were unable to comprehensively compare clinical variables across the TCGA, E-MTAB-1980, and GSE29609 cohorts, which represents an important limitation in assessing whether baseline confounders contributed to the observed survival discrepancy. Although the TNM stage distribution in GSE29609 was comparable to that in the TCGA cohort (**Supplementary Table 4**), the limited sample size rendered the risk stratification susceptible to unmeasured confounders. Thus, we believed that this discrepancy most likely reflected statistical underpowering rather than a true biological reversal of the prognostic value of our risk model, a conclusion further supported by the consistent validation results in the larger E-MTAB-1980 cohort.

In the present study, we ultimately selected 13 hub genes based on the C-index to construct a prognostic model. NR3C2, the mineralocorticoid receptor, is reportedly overexpressed in RCC cell proliferation, colony formation, invasion, migration, and vascular mimicry, reducing the growth of RCC xenografts *in vivo* (29). Furthermore, Yan et al. (30) discovered that miR-155-5p influenced ccRCC malignant progression by targeting NR3C2. However, its connection to lactylation-related modifications remains unexplored. Diminished KLF6 expression in renal cells promotes E2F1 transcription, thereby enhancing epithelial–mesenchymal transition and metastatic potential in ccRCC (31). As a transcription factor, E2F1 directly binds to the SMEK1 promoter as demonstrated by chromatin immunoprecipitation, inducing ccRCC progression by regulating SMEK1 expression (32). Beyond its pivotal role in cell cycle regulation, E2F1 also upregulates sterol regulatory element–binding protein 1, thereby inducing considerable lipid accumulation and elevated lipase levels in ccRCC cells (33). SETD2 is a histone H3 lysine 36 trimethyltransferase whose loss occurs in approximately 13% of ccRCC cases. This deficiency leads to increased chromatin binding of ASF1B, upregulates metastasis-associated genes, and thereby promotes ccRCC invasion and metastasis (34). The related methyltransferase SETD7 regulates another key molecule in our prognostic model, CCNA2, through the SETD7–TAF7–CCNA2 axis, further influencing ccRCC proliferation and metastatic progression (35). In summary, the roles of most hub genes in the lactylation-related gene model within ccRCC have been relatively well established. However, the specific regulatory relationships between these genes and lactylation remain to be further elucidated. Additionally, the biological function of CDT1 is of particular interest, as its biological role in ccRCC has not been previously reported.

CDT1 is a DNA replication licensing factor (36). In the G1 phase, it loads the MCM2–7 complex onto replication origins to ensure that DNA is replicated only once per cell cycle. In the S phase, CDT1 is rapidly degraded via ubiquitination by SCF-Skp2 or CRL4-Cdt2, thereby preventing re-replication. Dysregulation of CDT1 can lead to aberrant DNA re-replication and aneuploidy, which are closely associated with tumorigenesis and cellular senescence (37). Polyploid giant cancer cells (PGCCs), a distinct subtype of tumor cells, exhibit unique morphological characteristics. Pan et al. revealed that elevated CDT1 expression significantly induced PGCC formation, enhanced cancer stemness, and promoted chromosomal instability (38). CDT1 can activate the PLK4/SASS6 axis, thereby contributing to therapy resistance and malignant progression. Several CDT1 inhibitors have been identified to date, including simvastatin and the CDT1/geminin complex-targeting agent AF615, which has been shown to sensitize prostate cancer to PARP inhibitors (38, 39). Our study demonstrated the tumor-promoting role of CDT1 in ccRCC, highlighting its potential as a valuable molecular biomarker and therapeutic target. Nevertheless, the present data do not establish a direct causal link between CDT1 and lactylation. Whether CDT1 itself is lactylated or whether it regulates lactylation-dependent signaling requires further investigation using pan-Kla, site-specific lactylation assays, and lactylation-modulation experiments.

Lactate and lactylation are closely associated with immune cells (40). Lactate exerts immunosuppressive effects by regulating the functions of various immune cells, including natural killer cells, T cells, dendritic cells, and monocytes (41). Our lactylation-related gene model revealed differences in the proportions of T cells and M1 macrophages across distinct risk groups. Lactate suppresses histone deacetylase activity, leading to increased H3K27 acetylation at the Tcf7 super-enhancer site and subsequent upregulation of Tcf7 gene expression (42). Further, it enhances CD8 T cell pluripotency and boosts antitumor immunity. Compared with CD8^+^ T cells, CD4^+^ T cells exhibit markedly reduced migratory capacity in the presence of lactate, primarily through SLC5A12-mediated metabolic reprogramming (41). Lactate also enters CD4^+^ T cells through MCT1, upregulates mitochondrial LDHA, blocks Foxp3 ubiquitination, and ultimately increases the proportion of Foxp3^+^ Tregs, exerting an immunosuppressive effect on the immune microenvironment (43). In ccRCC, VHL inactivation drives hyperglycolysis, causing a dramatic increase in lactate accumulation in the tumor microenvironment. This metabolic remodeling promotes histone lactylation, induces an exhausted phenotype in CD8^+^ T cells, upregulates PD-1, suppresses effector molecules such as IFN-γ and TNF-α, and blocks NFAT nuclear translocation and TCR signaling (44). Given the key role of lactylation in shaping the immune microenvironment and modulating immune checkpoint expression, we further evaluated the predictive value of our lactylation-based risk model for immunotherapy response. Because of the scarcity of publicly available ccRCC immunotherapy cohorts, we used the IMvigor210 cohort (metastatic urothelial carcinoma treated with atezolizumab) as a surrogate for initial proof-of-concept validation, supported by the shared immunobiology and common mechanisms of response to PD-1/PD-L1 blockade between these two immunogenic tumors. Nevertheless, extrapolation across cancer types should be interpreted cautiously and validation in RCC-specific immunotherapy cohorts is warranted once available.

The findings of this study are encouraging; however, it had several limitations. First, the analysis relied on retrospective data and public datasets, lacking validation in large-scale prospective studies and multi-center cohorts. Second, although the drug predictions derived from the model exhibited potential clinical relevance, they remain to be experimentally verified. Third, lactylation is a PTM that cannot be directly inferred from mRNA expression alone; direct validation of lactylation levels using techniques, such as pan-Kla, H3K18la/H3K9la immunofluorescence, ChIP-qPCR, or lactate/lactylation modulation experiments, was beyond the scope of this bioinformatics study and warrants future experimental investigation. Furthermore, although we have preliminarily explored the tumor-promoting role of CDT1 in ccRCC, its specific lactylation mechanisms are still not fully understood. Future studies should not only further elucidate the role of CDT1 lactylation in ccRCC but also incorporate multi-omics data, particularly spatial transcriptomics, to systematically reveal the cellular and spatial heterogeneity associated with lactylation at single-cell resolution.

## Conclusions

Based on 364 lactylation-related genes and integrated single-cell transcriptomic data, this study applied 4 computational methods to systematically delineate molecular heterogeneity in patients with ccRCC. Machine learning algorithms identified a 13-gene prognostic signature, from which a risk-scoring model was developed and validated. This model systematically revealed pronounced differences between high- and low-risk patients in terms of tumor microenvironment and immune regulation. Further, *in vitro* and *in vivo* experiments provided the preliminary functional validation that CDT1, a key component of the model, promotes ccRCC progression. These findings characterize the transcriptomic heterogeneity associated with lactylation-related gene signatures in ccRCC and provide a foundation for future investigations into metabolic–epigenetic crosstalk and the development of therapeutic strategies.

## Data Availability

The original contributions presented in the study are included in the article/**Supplementary Material**. Further inquiries can be directed to the corresponding authors.

## Supplementary material

Supplementary Figure 1 Evaluation of the lactylation-related prognostic model. **(a)** Univariate and **(b)** multivariate regression analyses showing the relationship between lactylation-related gene model scores and OS. **(c)** Nomogram calibration (1/3/5-year OS). **(d)** Clinical utility assessment using decision curve analysis. **(e)** Nomogram integrating lactylation model with clinical parameters. **(f, g)** Ridge plots showing GO terms enriched in **(f)** high-risk and **(g)** low-risk groups.

Supplementary Figure 2 Transcriptome features and genetic alterations related to lactylation model. **(a)** Violin plot showing the difference in MATH scores between the high- and low-risk groups. **(b, c)** Kaplan–Meier survival analyses for OS based on MATH score alone or combined with the lactylation-related gene model risk score. **(d, e)** Waterfall plots of the somatic mutation landscape in **(d)** high-risk and **(e)** low-risk groups. **(f, g)** Co-occurrence and mutual exclusivity analysis of somatic mutations in **(f)** high-risk and **(g)** low-risk groups.

Supplementary Figure 3 Expression of 13 hub genes of the lactylation-related prognostic model in various cell types analyzed by single-cell data analysis.

Supplementary Table 1 364 genes related to lactylation compiled from previously published data.

Supplementary Table 2 Differentially expressed genes (DEGs) between high- and low-lactylation score groups in the single-cell transcriptome.

Supplementary Table 3 Module genes associated with lactylation-related scores identified by WGCNA.

Supplementary Table 4 Clinical information for 39 patients with ccRCC in the GSE29609 dataset.

Supplementary Table 5 Clinical and experimental sample information for 12 patients with ccRCC.
